# The Balance of Fluid and Osmotic Pressures across Active Biological Membranes with Application to the Corneal Endothelium

**DOI:** 10.1371/journal.pone.0145422

**Published:** 2015-12-31

**Authors:** Xi Cheng, Peter M. Pinsky

**Affiliations:** Department of Mechanical Engineering, Stanford University, Stanford, California, United States of America; UC Santa Barbara, UNITED STATES

## Abstract

The movement of fluid and solutes across biological membranes facilitates the transport of nutrients for living organisms and maintains the fluid and osmotic pressures in biological systems. Understanding the pressure balances across membranes is crucial for studying fluid and electrolyte homeostasis in living systems, and is an area of active research. In this study, a set of enhanced Kedem-Katchalsky (KK) equations is proposed to describe fluxes of water and solutes across biological membranes, and is applied to analyze the relationship between fluid and osmotic pressures, accounting for active transport mechanisms that propel substances against their concentration gradients and for fixed charges that alter ionic distributions in separated environments. The equilibrium analysis demonstrates that the proposed theory recovers the Donnan osmotic pressure and can predict the correct fluid pressure difference across membranes, a result which cannot be achieved by existing KK theories due to the neglect of fixed charges. The steady-state analysis on active membranes suggests a new pressure mechanism which balances the fluid pressure together with the osmotic pressure. The source of this pressure arises from active ionic fluxes and from interactions between solvent and solutes in membrane transport. We apply the proposed theory to study the transendothelial fluid pressure in the *in vivo* cornea, which is a crucial factor maintaining the hydration and transparency of the tissue. The results show the importance of the proposed pressure mechanism in mediating stromal fluid pressure and provide a new interpretation of the pressure modulation mechanism in the *in vivo* cornea.

## Introduction

The exchange of fluid and solutes across biological membranes facilitates the transport of substances needed for living organisms to maintain their metabolic activities, and regulates pressure balances across bounding membranes to maintain the structural integrity of biological systems. The movement of these substances is controlled by both passive and active transport processes. Passive transport mechanisms drive water or solutes to move down their concentration gradients without need of energy input, whereas active transport mechanisms propels solutes to move against their concentration gradients at the cost of energy input from metabolic reactions. The interplay between the two mechanisms determines the fluid hydrostatic pressure and osmotic pressure differences across biological membranes, which are important characteristics for biological systems. For example, at the organ level, fluid pressure mediates fluid transport between capillaries and tissues, and facilitates the diffusion of nutrients between the two compartments [1]. At the cellular level, fluid pressure interacts with osmotic pressure to regulate cell volumes at normal state [2, 3] and to drive its shape changes during processes such as protrusion and blebbing [4, 5]. A disturbance of the pressure regulation mechanisms can lead to swelling or shrinking of cells and tissues. A quantitative understanding of fluid and osmotic pressures in living organisms is crucial for studying biological mechanisms such as cell volume regulation and interstitial fluid homeostasis, and is under investigation for various biological systems, e.g. [2, 3, 6, 7]. The goal of the present work is to develop a mathematical description of water and solute transport across membranes and apply it to study the pressure balance conditions in biological systems, characterizing passive and active transport mechanisms and other biological features.

Cells and connective tissues contain a significant concentration of negatively charged molecules (proteins, organic phosphates or sulphated proteoglycans) to which the bounding membrane is impermeable [3, 8]. The presence of fixed charges attracts counterions and produces an osmolarity that is higher in the internal compartment than in the external compartment, which is separated from the internal compartment by the membrane. As a result, an osmotic pressure gradient is developed which drives water to flow into the interior compartment and thereby cause swelling. In many physiological systems, the above noted swelling tendency is counterbalanced by active ionic transport processes that are located in bounding membranes and which produce outward fluxes of ions that can modulate the osmotic pressure induced by fixed charges [3]. In animal cells, the maintenance of cell volumes relies heavily on the pressure balance mediated by active ionic transport processes and intracellular fixed charges [2, 3].

In order to describe transport phenomena in such biological systems, nonequilibrium thermodynamics has been employed to characterize the relationship between fluid and ionic fluxes and their generalized driving forces [9–11], with the latter originating in the gradient of the electrochemical potential and affinity of metabolic reactions. The theory is predicated on the existence of a dissipation function which describes the total change of system entropy. For near-equilibrium systems, in which the rate of free energy dissipation is small (i.e. the system is “is not too far” from equilibrium), linear relationships between fluxes and the driving forces can be assumed [9, 11]. The classical Kedem-Katchalsky (KK) theory [12] was developed along these lines. KK theory takes account of interactions between solvent and non-ionic solutes, and results in a set of phenomenological membrane coefficients that are readily evaluated experimentally. This theory provides the theoretical foundations for analyzing fluid flow and solute fluxes of various membrane systems.

Because the original KK theory was limited to passive transport in nonelectrolytes, numerous extensions and modifications have been proposed to include additional biophysical effects. Kedem and Katchalsky [13] extended the original KK equations to study the transport of a binary electrolyte through charged membranes. In addition to consideration of water flow and ionic fluxes, the effect of electric currents has been incorporated into KK theory to describe electroosmosis phenomena. Hoshiko and Lindley [14] integrated active ionic transport mechanisms into the KK equations for single salt and bi-ionic systems. This work developed a convenient approach to describe active movement of ions in a membrane system without the need to know the underlying detailed molecular mechanisms. Kargol [15] proposed a set of modified KK equations that takes into account the effect of boundary layers on the passive transport across a membrane. The modified theory is shown to give better prediction for glucose flux across a nephrophane membrane. More recently, Li [16] derived a new set of KK equations for the transport of multiple ionic species across membranes. This work derived a new volume flux formulation which includes an additional driving force that originates from the transmembrane electrostatic potential difference.

While these developments of the KK theory have covered a wide range of biophysical conditions, the effect of fixed charges has been neglected; KK theories to date have only considered mobile ions and employed the electroneutrality assumption (i.e. Σ_*i*_ *z*_*i*_ *C*_*i*_ = 0). In the current study, we evaluate the influence of fixed charges on fluid and solutes transport across biological membranes. First we show that due to neglect of fixed charges, existing KK theories, including [12, 16], predict zero fluid pressure difference across membranes at thermodynamic equilibrium. We then employ linear nonequilibrium thermodynamics and propose a set of enhanced KK equations considering: 1) the presence of fixed charges on one side of a membrane, 2) transport of both ionic and non-ionic species and 3) active ionic transport mechanisms which move solutes from a lower to a higher concentration region. The proposed theory is capable of recovering the Donnan equilibrium and predicting the correct fluid pressure that is required to balance the Donnan osmotic pressure at equilibrium state. The analysis explains the swelling tendency of a charged electrolyte gel regardless of the presence of bounding layers.

In addition to illustrating the fixed charge effect, we apply the proposed KK equations to study the water transport across active biological membranes, which was believed to be governed by the balance of osmotic pressure and fluid pressure [3]. Our analysis identifies an additional pressure mechanism that originates from active fluxes and from interactions between water and solutes in membrane transport processes. This pressure force competes with the osmotic pressure on balancing the fluid pressure, and the new pressure balance condition implies that the values of the water potential on the two sides of separating membranes will not be equal in order to maintain the steady state of biological systems. To illustrate the importance of this new pressure mechanism, we apply the enhanced KK equations to quantify the transendothelial fluid pressure in the *in vivo* cornea, in which active transport mechanisms play crucial roles in regulating the fluid transport across the corneal endothelial layer. The results show that the additional pressure mechanism has a significant impact on influencing the fluid pressure.

## Limitations of existing Kedem-Katchalsky theories

**Fig 1:**
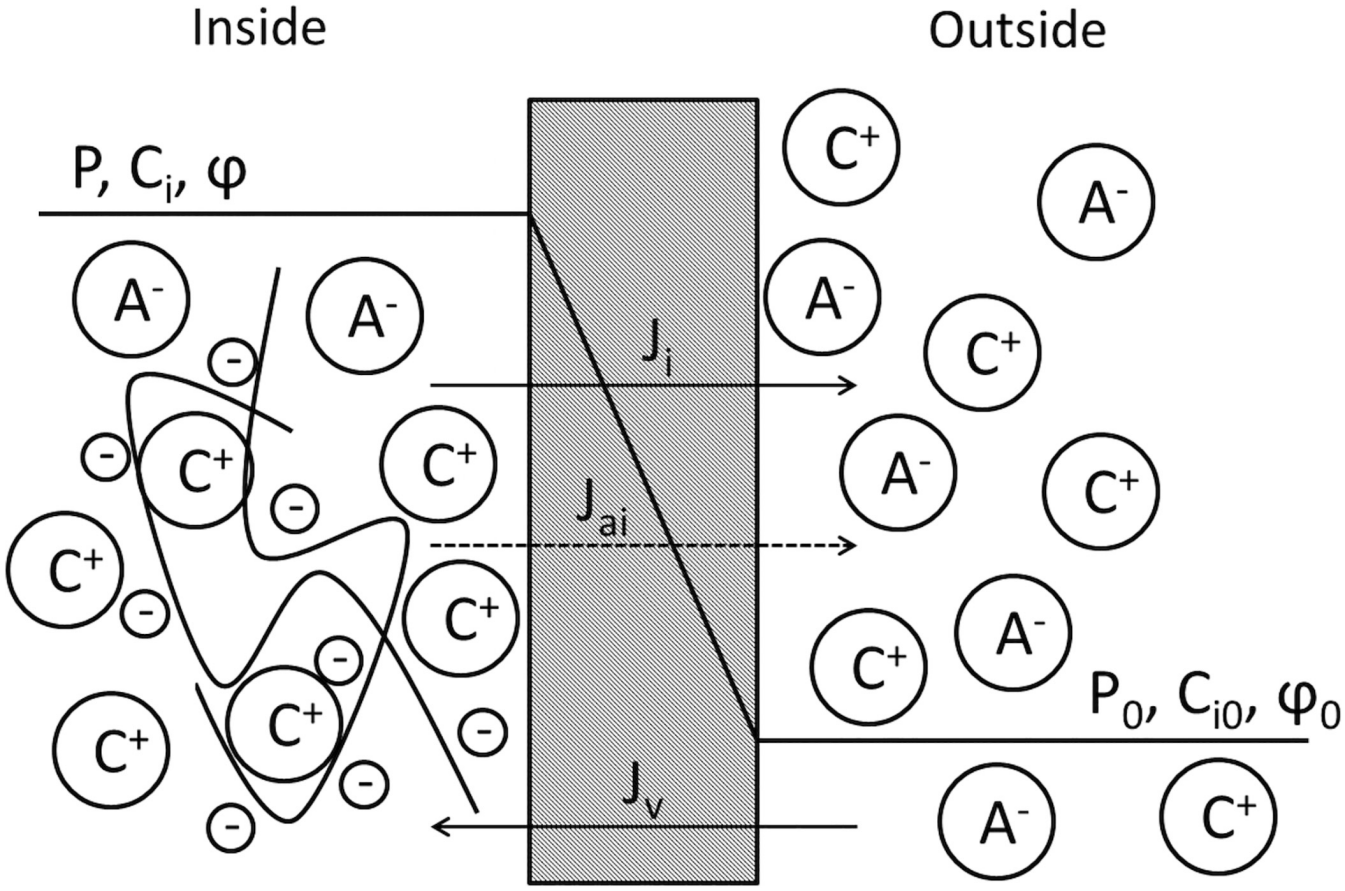
Illustration of a biological membrane that separates two electrolyte solutions, with the one designated as “inside” containing fixed charges that are associated with large molecules.

In this section we describe a limitation of existing KK theories [12, 16] which are unable to recover the Donnan equilibrium when fixed charges exist on one side of the membrane. Consider a biological membrane that separates two polyelectrolyte solutions with fluid pressure *P* and *P*_0_, solute concentrations *C*_*i*_ and $C_{i}^{0}\left(i=1,...,N\right)$, where *N* denotes the number of species, and electrostatic potential *φ* and *φ*_0_. We denote one side of the membrane as “inside” and the other as “outside” (see Fig 1) and assume the inside electrolyte solution contains large molecules that carry fixed charges with concentration *C*_f_ and valence value *z*_f_. The fixed charges are assumed to be “trapped” in the inside solution and the biological membrane is assumed to be impermeable to large molecules [3]. Both solvent and solutes are considered to have finite permeabilities through the membrane (i.e. the membrane is leaky). The classical KK equations [12] describe the volume flux *J*_*V*_ and solute flux *J*_*i*_(*i* = 1, …, *N*) between the two solutions as follows:

$$\begin{array}{cc} J_{V} & =L_{p}\left(\Delta P-\sum_{k}\sigma_{k}RT\Delta C_{k}\right) \end{array}$$ (1)

$$\begin{array}{cc} J_{i} & =\left(1-\sigma_{i}\right)J_{V}{\bar{C}}_{i}+\omega_{i}RT\Delta C_{i} \end{array}$$ (2)

where *L*_*p*_ is the hydraulic conductivity, *σ*_*i*_ and *ω*_*i*_ are the reflection coefficient and permeability for species *i*, respectively, and Δ*P* and Δ*C*_*i*_ are the fluid pressure difference and ionic concentration difference across the membrane, respectively. ${\bar{C}}_{i}$ denotes the mean ionic concentration, and can be simplified as the arithmetic mean between *C*_*i*_ and $C_{i}^{0}$ (i.e. ${\bar{C}}_{i}=\left(C_{i}+C_{i}^{0}\right)/2$). Consider the equilibrium condition in which no fluid flow and no ionic fluxes exist across the membrane, i.e. *J*_*V*_ = *J*_*i*_ = 0, Eqs (1, 2) immediately give

$$\begin{array}{cc} \Delta C_{i} & =0 \end{array}$$ (3)

$$\begin{array}{cc} \Delta P & =0 \end{array}$$ (4)

which suggests that at equilibrium, ionic concentrations will be balanced and there will be no fluid pressure difference across the membrane. This conclusion is apparently contradicted by the well-known Donnan equilibrium where fixed charges induce imbalance of ionic concentrations and develop an osmotic pressure gradient between the inside and outside environments [17]. This limitation of Eqs (1, 2) is attributed to the fact that they were developed for transport of non-ionic species [12]. Li [16] derived an extended set of KK equations which incorporate the electrostatic potential difference between separated electrolyte solutions,

$$\begin{array}{cc} J_{V} & =L_{p}\left(\Delta P-\sum_{k}\sigma_{k}\left(RT\Delta C_{k}+z_{k}F{\bar{C}}_{k}\Delta \varphi \right)\right) \end{array}$$ (5)

$$\begin{array}{cc} J_{i} & =\left(1-\sigma_{i}\right)J_{V}{\bar{C}}_{i}+\omega_{i}\left(RT\Delta C_{i}+z_{i}{\bar{C}}_{i}F\Delta \varphi \right) \end{array}$$ (6)

The above equations are still unable to recover the fluid pressure difference corresponding to Donnan equilibrium. At the equilibrium state (with *J*_*V*_ = *J*_*i*_ = 0), Eqs (5, 6) imply zero pressure difference and nonzero ionic concentrations difference, which suggests a nonphysical situation. Regardless of the ionic concentrations difference across the membrane due to the fixed charges, no fluid pressure difference is needed to balance the resulting osmotic pressure difference.

The inconsistency between Eqs (5, 6) and the Donnan theory is associated with the neglect of the fixed charges. Presently, the only KK theory that incorporates the fixed charge effect is the one proposed by Hodson and Earlam [18] considering binary solutions:

$$\begin{array}{cc} J_{V} & =L_{p}\left((\Delta P-\sigma \Delta \Pi )-(1-\sigma )\Delta \gamma \right) \end{array}$$ (7)

$$\begin{array}{cc} J_{i} & =\left(1-\sigma \right)J_{V}C_{0}+\frac{\omega }{\sigma }\left(\Delta P-\Delta \gamma \right) \end{array}$$ (8)

where *σ* and *ω* are the membrane transport coefficients, *C*_0_ is the binary electrolyte concentration of the outside solution, ΔΠ = *RT*(*C*_+_+*C*_−_−2*C*_0_) is the osmotic pressure, and $\Delta \gamma =RTC_{\text{f}}^{2}/4C_{0}$ is called the gel pressure and is a function of fixed charge concentration. Interestingly, applying the zero flux conditions to Eqs (7, 8) gives Δ*P* = Δγ = ΔΠ, although the theory is only feasible for binary solutions and no active transport is considered. In this study, we employ linear non-equilibrium thermodynamics and derive a modified form of the KK equations for a polyelectrolyte solution with fixed charges. We then further extend the theory to include active ion transport mechanisms.

## Modified KK equations for passive transport

Consider a leaky membrane that separates two electrolyte solutions as described above. The movement of solvent and solutes across the membrane can be characterized by a set of fluxes and conjugate forces according to nonequilibrium thermodynamics [11]. The identifications of these quantities are based on the statement of dissipation function Φ, which describes the rate that the free energy is dissipated during transport. Its mathematical formulation is given as [9, 10]

$$\begin{array}{c} \Phi =J_{w}X_{w}+\sum_{i=1}^{N}J_{i}X_{i} \end{array}$$ (9)

where *J*_*w*_ and *J*_*i*_ (*i* = 1, …, *N*) denote fluid flow and solute fluxes, respectively, and *X*_*w*_ and *X*_*i*_ denote the corresponding conjugate forces for fluid and solutes. In their simplest form, *X*_*w*_ and *X*_*i*_ can be written as the electrochemical potential difference across the membrane [10, 12], i.e.

$$\begin{array}{cc} X_{w} & =\Delta \mu_{w} \end{array}$$ (10)

$$\begin{array}{cc} X_{i} & =\Delta \mu_{i} \end{array}$$ (11)

where *μ*_*w*_ and *μ*_*i*_ are the electrochemical potential for fluid and solute species *i*, respectively. In order to derive the mathematical forms for Δ*μ*_*w*_ and Δ*μ*_*i*_, we first recall the electrochemical potential for water and ions as:

$$\begin{array}{cc} \mu_{w} & =\nu_{w}\left(P-RT\sum_{k}C_{k}\right) \end{array}$$ (12)

$$\begin{array}{cc} \mu_{i} & =\nu_{i}P+RT\ln C_{i}+z_{i}F\varphi \end{array}$$ (13)

where *ν*_*w*_ and *ν*_*i*_ in m^3^/mol are the partial volume of solvent and solutes, respectively, *z*_*i*_ is the valence number for species *i*, *R*, *T* and *F* are gas constant, temperature and Faraday constant, respectively. The linearized forms for Δ*μ*_*w*_ and Δ*μ*_*i*_ are then given as

$$\begin{array}{cc} \Delta \mu_{w} & =\nu_{w}\left(\Delta P-RT\sum_{k}\Delta C_{k}\right) \end{array}$$ (14)

$$\begin{array}{cc} \Delta \mu_{i} & =\nu_{i}\Delta P+RT\frac{\Delta C_{i}}{{\bar{C}}_{i}}+z_{i}F\Delta \varphi \end{array}$$ (15)

where ${\bar{C}}_{i}=\left(C_{i}+C_{i}^{0}\right)/2$ is the average ionic concentration through the membrane.

Following a common convention [12, 16], we rewrite the dissipation function in terms of a new set of forces and fluxes that are easier to assess experimentally

$$\begin{array}{ll} \Phi & =J_{w}\Delta \mu_{w}+\underset{i}{\sum^{\;}}J_{i}\Delta \mu_{i}\\ & =\left(\nu_{w}J_{w}+\underset{k}{\sum^{\;}}\nu_{k}J_{k}\right)\Delta \tilde{P}+\underset{i}{\sum^{\;}}\;\left(\frac{J_{i}}{{\bar{C}}_{i}}-\frac{J_{w}}{C_{w}}\right)\Delta {\tilde{\Pi }}_{i}\\ & =J_{V}\Delta \tilde{P}+\underset{i}{\sum^{\;}}J_{Di}\Delta {\tilde{\Pi }}_{i} \end{array}$$ (16)

where *C*_*w*_ is the solvent (water) concentration, *J*_*V*_ = *ν*_*w*_ *J*_*w*_ + ∑_*k*_ *ν*_*k*_ *J*_*k*_ is the volume flux across the membrane, and $J_{Di}=J_{i}/{\bar{C}}_{i}-J_{w}/C_{w}$ is the exchange flux for species *i* and can be interpretated as the relative velocity of the solute versus the solvent. The corresponding conjugate forces $\Delta \tilde{P}$ and $\Delta {\tilde{\Pi }}_{i}$ are given as

$$\begin{array}{cc} \Delta \tilde{P} & =\left(C_{w}\nu_{w}+\sum_{k}C_{k}\nu_{k}\right)\Delta P+RT\left(1-C_{w}\nu_{w}\right)\sum_{k}\Delta C_{k}+RT\sum_{k}z_{k}F{\bar{C}}_{k}\Delta \varphi \end{array}$$ (17)

$$\begin{array}{cc} \Delta {\tilde{\Pi }}_{i} & =RT\Delta C_{i}+z_{i}F{\bar{C}}_{i}\Delta \varphi -{\bar{C}}_{i}\nu_{i}\sum_{k}z_{k}F{\bar{C}}_{k}\Delta \varphi \end{array}$$ (18)

Note that the sum of all volume fractions is unity, i.e.

$$\begin{array}{c} C_{w}\nu_{w}+\sum_{k}C_{k}\nu_{k}=1 \end{array}$$ (19)

for dilute solutions, the solvent (water) volume fraction is dominant over that of solutes, and therefore is close to one, i.e. *C*_*w*_ *ν*_*w*_ ≈ 1. Eqs (17) and (18) can then be simplified as

$$\begin{array}{cc} \Delta \tilde{P} & =\Delta P+RT\sum_{k}z_{k}F{\bar{C}}_{k}\Delta \varphi \end{array}$$ (20)

$$\begin{array}{cc} \Delta {\tilde{\Pi }}_{i} & =RT\Delta C_{i}+z_{i}F{\bar{C}}_{i}\Delta \varphi \end{array}$$ (21)

where Eq (20) describes the effects of fluid pressure and electrostatic potential on the volume flux, and is a more general expression compared to that given in [16], which eliminates the second term using an electroneutrality condition of the form ∑_*k*_ *z*_*k*_ *C*_*k*_ = 0 (i.e. neglecting the fixed charges). The new formulation (20) will lead to a set of modified KK equations which predict the correct fluid pressure in Donnan equilibrium.

Employing linear nonequilibrium thermodynamics [9, 10], the volume flux *J*_*V*_ and the exchange flux *J*_*Di*_ are assumed to be linear functions of the conjugate forces $\Delta \tilde{P}$ and $\Delta {\tilde{\Pi }}_{i}$,

$$\begin{array}{cc} J_{V} & =L_{VV}\Delta \tilde{P}+\sum_{k}L_{Vk}\Delta {\tilde{\Pi }}_{k} \end{array}$$ (22)

$$\begin{array}{cc} J_{Di} & =L_{iV}\Delta \tilde{P}+\sum_{k}L_{Dik}\Delta {\tilde{\Pi }}_{k} \end{array}$$ (23)

where *L*_*Vk*_ = *L*_*kV*_, *k* = 1, …, *N* and *L*_*Dij*_ = *L*_*Dji*_, *i*, *j* = 1, …, *N*, satisfying the Onsager reciprocal relation Schultz1980. Rewriting the above equations as

$$\begin{array}{cc} J_{V} & =L_{p}\Delta \tilde{P}-L_{p}\sum_{k}\sigma_{k}\Delta {\tilde{\Pi }}_{k} \end{array}$$ (24)

$$\begin{array}{cc} J_{Di} & =-L_{p}\sigma_{i}\Delta \tilde{P}+\sum_{k}L_{Dik}\Delta {\tilde{\Pi }}_{k} \end{array}$$ (25)

where *L*_*p*_ and *σ*_*k*_ are the hydraulic conductivity coefficients and reflection coefficient that have the following formal definition:

$$\begin{array}{cc} L_{p} & \equiv {\left(\frac{J_{V}}{\Delta \tilde{P}}\right)}_{\Delta {\tilde{\Pi }}_{k}=0}=L_{VV} \end{array}$$ (26)

$$\begin{array}{cc} \sigma_{i} & \equiv -\frac{L_{Vi}}{L_{VV}} \end{array}$$ (27)

Assuming a dilute solution, the solute flux *J*_*i*_ can be expressed as [10]:

$$\begin{array}{l} \begin{array}{cc} J_{i} & =\left(J_{V}+J_{Di}\right){\bar{C}}_{i} \end{array}\\ \begin{array}{cc} & \;=\left(1-\sigma_{i}\right)J_{V}{\bar{C}}_{i}+\underset{k}{\sum^{\;}}\omega_{ik}\Delta {\tilde{\Pi }}_{k} \end{array} \end{array}$$ (28)

where $\omega_{ik}={\bar{C}}_{i}\left(L_{Dik}-L_{p}\sigma_{i}\sigma_{k}\right)$ denotes the permeability coefficients. Ignoring the interactions between ionic fluxes (i.e. *ω*_*ik*_ = 0 for *i* ≠ *k*) and denote *ω*_*i*_ = *ω*_*ii*_,

$$\begin{array}{c} J_{i}=\left(1-\sigma_{i}\right)J_{V}{\bar{C}}_{i}+\omega_{i}\Delta {\tilde{\Pi }}_{i} \end{array}$$ (29)

where *ω*_*i*_ has the following formal definition

$$\begin{array}{c} \omega_{i}\equiv {\left(\frac{J_{i}}{\Delta {\tilde{\Pi }}_{i}}\right)}_{J_{V}=0} \end{array}$$ (30)

In summary, the volume flux *J*_*V*_ and solute flux *J*_*i*_ can be written as a linear combination of Δ*P*, Δ*C*_*i*_ and Δ*φ* by substituting Eqs (20, 21) into Eqs (24, 29):

$$\begin{array}{cc} J_{V} & =L_{p}\left(\Delta P-\sum_{k}\left(\sigma_{k}RT\Delta C_{k}-\left(1-\sigma_{k}\right)z_{k}F{\bar{C}}_{k}\Delta \varphi \right)\right) \end{array}$$ (31)

$$\begin{array}{cc} J_{i} & =\left(1-\sigma_{i}\right)J_{V}{\bar{C}}_{i}+\omega_{i}\left(RT\Delta C_{i}+z_{i}{\bar{C}}_{i}F\Delta \varphi \right) \end{array}$$ (32)

It should be noted that the volume flux expression Eq (31) differs from Eq (5) by the addition of the term $-\sum_{k}z_{k}F{\bar{C}}_{k}\Delta \phi$, which is essentially zero in Li [16] due to neglect of the fixed charge concentration. The effect of this correction term will be discussed in the following section.

## Application to equilibrium conditions: recovery of Donnan equilibrium

In this section the modified KK Eqs (31) and (32) are used to study the Donnan equilibrium in which unequal distributions of ionic concentration and fluid pressure are developed between two ionic solutions separated by a membrane [17]. At thermodynamic equilibrium, no macroscopic flow of fluid or solutes occurs between the two solutions, i.e. *J*_*V*_ = *J*_*i*_ = 0, and the dissipation function is zero (see Eq (9)). Applying the KK theory to study this classical condition provides the baseline for the study of the effects of active fluxes. We show that the new theory recovers the Donnan osmotic pressure, and predicts the correct fluid pressure that is required to balance the osmotic pressure. The predicted pressure quantities do not depend on membrane transport properties, indicating that the Donnan equilibrium will be satisfied regardless of the presence of a biological membrane [18].

At equilibrium, the zero flux conditions *J*_*V*_ = 0 and *J*_*i*_ = 0 requires

$$\begin{array}{c} \omega_{i}\left(RT\Delta C_{i}+z_{i}{\bar{C}}_{i}F\Delta \varphi \right)=0 \end{array}$$

which implies

$$\begin{array}{c} -\left(1-\sigma_{i}\right)z_{i}{\bar{C}}_{i}F\Delta \varphi =\left(1-\sigma_{i}\right)RT\Delta C_{i} \end{array}$$ (33)

The equilibrium fluid pressure difference Δ*P*, derived from *J*_*V*_ = 0, is then

$$\begin{array}{ll} \Delta P & =\underset{k}{\sum^{\;}}\left(\sigma_{k}RT\Delta C_{k}-\left(1-\sigma_{k}\right)z_{k}F{\bar{C}}_{k}\Delta \varphi \right)\\ & =\underset{k}{\sum^{\;}}\sigma_{k}RT\Delta C_{k}+\left(1-\sigma_{k}\right)RT\Delta C_{k}\\ & =RT\underset{k}{\sum^{\;}}\Delta C_{k} \end{array}$$ (34)

From Eq (34) it can be seen that Δ*P* = *RT*∑_*k*_ Δ*C*_*k*_ = ΔΠ is satisfied independently of the membrane properties (*σ*_*i*_, *ω*_*i*_), indicating that regardless of whether the membrane is nearly semipermeable (*σ*_*k*_ → 1) or completely freely permeable (*σ*_*k*_ → 0), the equilibrium fluid pressure difference always equals the osmotic pressure difference across the membrane.

Next we derive the mathematical formulation for Δ*C*_*k*_ and show that Δ*P* equals the Donnan osmotic pressure. Consider the separated solutions shown in Fig 1 as binary solutions, and assume the valence value of the fixed charges as *z*_f_ = −1. The sign convention is given as follows: net fluxes of fluid and solutes from the inside to the outside solution are taken as positive, and Δ(⋅) is calculated by the quantity in the inside minus that in the outside. The equilibrium conditions *J*_+_ = *J*_−_ = 0 then give:

$$\begin{array}{cc} RT\left(C_{+}-C_{0}\right)+\frac{C_{0}+C_{+}}{2}F\Delta \varphi & =0 \end{array}$$ (35)

$$\begin{array}{cc} RT\left(C_{-}-C_{0}\right)-\frac{C_{0}+C_{-}}{2}F\Delta \varphi & =0 \end{array}$$ (36)

where $C_{0}=C_{+}^{0}=C_{-}^{0}$ is the bath concentration. The above equations result in

$$\begin{array}{c} C_{+}C_{-}=C_{0}^{2} \end{array}$$ (37)

which is the Donnan equilibrium condition [17]. Using the electroneutrality condition for the electrolyte, including the fixed charge,

$$\begin{array}{c} C_{+}=C_{-}+C_{\text{f}} \end{array}$$ (38)

the cation and anion concentrations can be solved from Eqs (37, 38), and Δ*P* is given as:

$$\begin{array}{ll} \Delta P & =RT\left(C_{+}+C_{-}-2C_{0}\right)\\ & =2RTC_{0}\left(\sqrt{\frac{C_{\text{f}}^{2}}{4C_{0}^{2}}+1}-1\right) \end{array}$$ (39)

which recovers the Donnan osmotic pressure [19]. The estimation of Δ*P* has important implications for membrane systems, since the fluid pressure difference corresponds to a mechanical force difference applied at the membrane. Positive Δ*P* indicates an expansion (swelling) tendency for inside of the membrane, and vice versa. It governs the volume of the interior compartment from bounding membranes, for example in cells [2], in vesicles [20] and in the human cornea [21].

## Extension to include the effect of active ionic transport

Active transport across cell membranes enables solute movement against their concentration gradient and is one of the major factors for keeping homeostasis within the body. It is divided into two types, according to the source of energy used, called primary active transport and secondary active transport. In the former category, energy is directly provided by the breakdown of adenosine triphosphate (ATP). In the latter category, energy is derived indirectly from energy stored in the form of ionic concentration differences between the two sides of a membrane. Directly modeling active mechanisms requires identifications of the reaction kinetics, which is only known for a few processes [22, 23]. Alternatively, active mechanisms can be incorporated into the nonequilibrium thermodynamic description of fluid and ionic transport by introducing the affinity of the driving metabolic reaction and its conjugate flux, the rate of reaction per unit membrane area [10, 14] (see details in S1 Appendix A). In the simplest form, the active ionic flux is treated as an independent term that is additive to the passive solute flux equation Eq (32). The net solute flux equation can then be written as

$$\begin{array}{c} J_{i}=\left(1-\sigma_{i}\right)J_{V}{\bar{C}}_{i}+\omega_{i}\left(RT\Delta C_{i}+z_{i}{\bar{C}}_{i}F\Delta \varphi \right)+J_{ai} \end{array}$$ (40)

It is noted that Eq (40) recovers the solute flux equation as used in [21, 24, 25] to describe the effect of active ionic flux without considering the underlying molecular mechanisms.

Similar to the case of thermodynamic equilibrium, we apply the extended KK Eqs (31, 40) to study the zero flux conditions for *J*_*V*_ and *J*_*i*_. As active transport mechanisms are presented and consume energy, the zero flux conditions correspond to a non-equilibrium steady state [26]. In this case, the fluid pressure difference Δ*P* across the membrane is derived by first observing from Eq (31) with *J*_*V*_ = 0 and Eq (32) with *J*_*i*_ = 0, that

$$\begin{array}{c} \omega_{i}\left(RT\Delta C_{i}+z_{i}{\bar{C}}_{i}F\Delta \varphi \right)+J_{ai}=0 \end{array}$$

which implies

$$\begin{array}{c} -\left(1-\sigma_{i}\right)z_{i}{\bar{C}}_{i}F\Delta \varphi =\left(1-\sigma_{i}\right)RT\Delta C_{i}+\frac{1-\sigma_{i}}{\omega_{i}}J_{ai} \end{array}$$ (41)

Now *J*_*V*_ = 0 implies

$$\begin{array}{c} \Delta P=\sum_{i}\left(RT\Delta C_{i}+\frac{1-\sigma_{i}}{\omega_{i}}J_{ai}\right)=\Delta \Pi +P_{\sigma } \end{array}$$ (42)

where ΔΠ = *RT*∑_*i*_ Δ*C*_*i*_ denotes the osmotic pressure and *P*_*σ*_ = ∑_*i*_(1−*σ*_*i*_)*J*_*ai*_/*ω*_*i*_ denotes an additional pressure force originating from the active ionic transport when the membrane is leaky (*σ*_*i*_ < 1).

The effect of *P*_*σ*_ on the fluid pressure difference Δ*P* may be studied by considering two binary electrolyte solutions, as described above, with an active anion flux *J*_*a*−_. For convenience, we denote *J*_*a*_ = *J*_*a*−_, *ω* = *ω*_Cl^−^_ and *σ* = *σ*_−_. Eqs (35, 36) then become

$$\begin{array}{cc} RT\left(C_{+}-C_{0}\right)+\frac{C_{0}+C_{+}}{2}F\Delta \varphi & =0 \end{array}$$ (43)

$$\begin{array}{cc} RT\left(C_{-}-C_{0}\right)-\frac{C_{0}+C_{-}}{2}F\Delta \varphi +\frac{J_{a}}{\omega } & =0 \end{array}$$ (44)

Solving Eqs (43), (44) and (38), the cation and anion concentrations are given as

$$\begin{array}{cc} C_{+} & =\frac{C_{\text{f}}}{2}-\frac{J_{a}}{4\omega RT}+\sqrt{{\left(\frac{C_{\text{f}}}{2}-\frac{J_{a}}{4\omega RT}\right)}^{2}-\frac{J_{a}}{2\omega RT}C_{0}+C_{0}^{2}} \end{array}$$ (45)

$$\begin{array}{cc} C_{-} & =-\frac{C_{\text{f}}}{2}-\frac{J_{a}}{4\omega RT}+\sqrt{{\left(\frac{C_{\text{f}}}{2}-\frac{J_{a}}{4\omega RT}\right)}^{2}-\frac{J_{a}}{2\omega RT}C_{0}+C_{0}^{2}} \end{array}$$ (46)

and the steady-state Δ*P* is then obtained from Eq (42) as:

$$\begin{array}{c} \Delta P=2RTC_{0}\left(\sqrt{{\left(\frac{C_{\text{f}}}{2C_{0}}-\frac{J_{a}}{4\omega RTC_{0}}\right)}^{2}-\frac{J_{a}}{2\omega RTC_{0}}+1}-\left(\frac{\sigma }{2}-\frac{1}{4}\right)\frac{J_{a}}{\omega RTC_{0}}-1\right) \end{array}$$ (47)

It can be seen that there are three free parameters controlling Δ*P*: the fixed charge concentration *C*_f_, the active ionic flux divided by the membrane permeability *J*_*a*_/*ω* and the reflection coefficient *σ*. The effects of these quantities on Δ*P* are discussed as follows.

**Fig 2:**
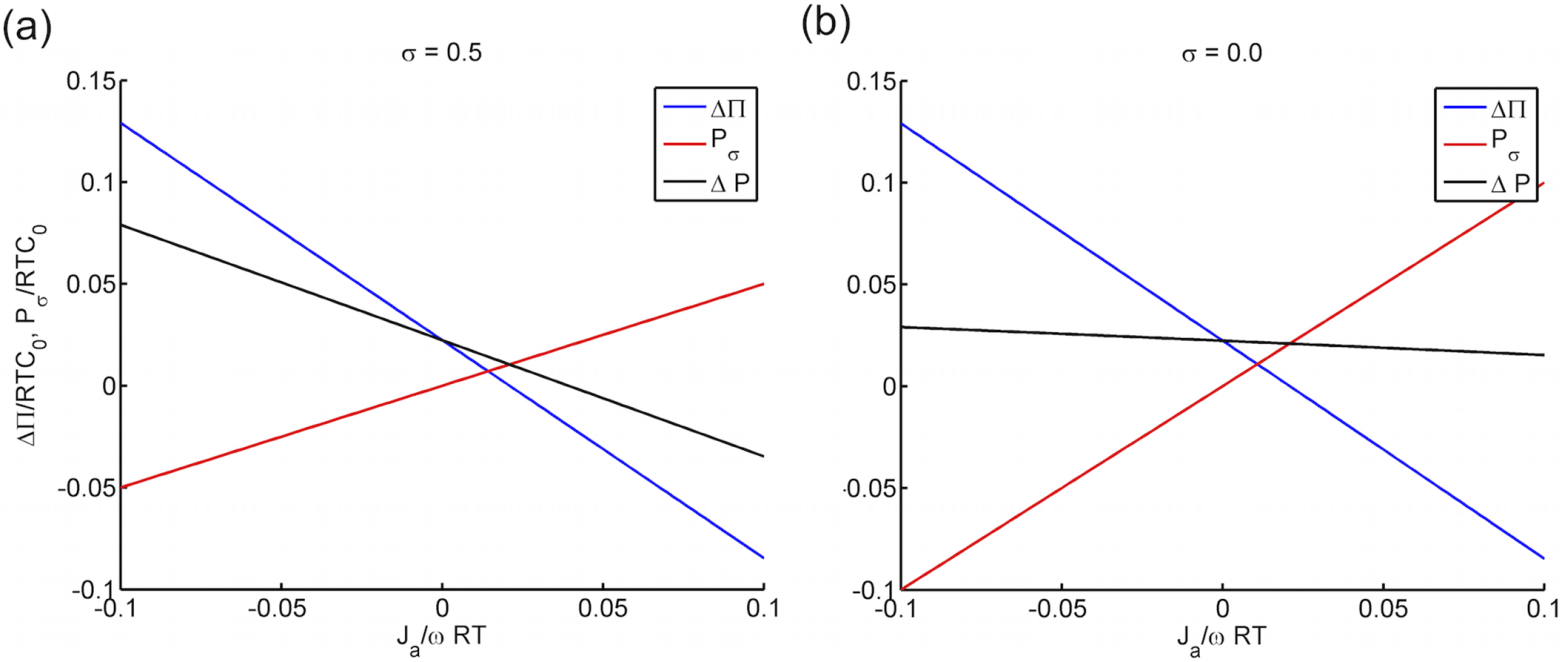
The predicted uid pressure difference Δ*P*, osmotic pressure difference ΔΠ and the additional pressure force *P*_*σ*_ against active ionic flux *J*_a_ for a) a leaky membrane with *σ* = 0.5 and b) a freely permeable membrane (*σ* = 0.0).

The pressure, fixed charge density and ionic fluxes are expressed in dimensionless forms as Δ*P*/*RTC*_0_, *C*_f_/*C*_0_ and *J*_*a*_/*ωRTC*_0_, respectively. The effect of active ionic flux on *P*_*σ*_, ΔΠ and Δ*P* are summarized in Fig 2 over the range of *J*_*a*_/*ωRTC*_0_ ∈ [−0.1, 0.1] at a fixed charge density *C*_f_/*C*_0_ = 0.3. Two values of *σ*, one representing a leaky membrane (*σ* = 0.5) and the other representing a freely permeable membrane (*σ* = 0.0), have been selected to illustrate the effect of *P*_*σ*_ on Δ*P*. An essentially linear relationship is observed between the active ionic flux and the three pressure estimations. The osmotic pressure ΔΠ is reduced when the active ionic flux is directed from inside to outside as described by positive *J*_*a*_. On the contrary, predicted *P*_*σ*_ increases with *J*_*a*_. The fluid pressure Δ*P*, which results from the two competing terms, decreases with *J*_*a*_, indicating that the active ionic transport has a stronger effect on ΔΠ than on *P*_*σ*_. Notably, the influence of *P*_*σ*_ on the fluid pressure difference is significant, especially when the membrane is more permeable (as described by small *σ*) to the active species. For *σ* = 0.5, the ratio between *P*_*σ*_ and ΔΠ is about 0.6 at *J*_*a*_/*ωRTC*_0_ = 0.1 (see Fig 2a). For a membrane that is freely permeable (*σ* = 0.0), the corresponding ratio increases to 1.2, resulting in a fluid pressure curve that is nearly unaffected by *J*_*a*_ (see Fig 2b). If the membrane is nearly semipermeable (*σ* → 1.0), *P*_*σ*_ is zero and has no effect on Δ*P*. The results in Fig 2 show that the pressure force *P*_*σ*_ plays a significant role in countering the active ionic transport effect on the fluid pressure.

## Application to endothelial transport in the *in vivo* cornea

**Fig 3:**
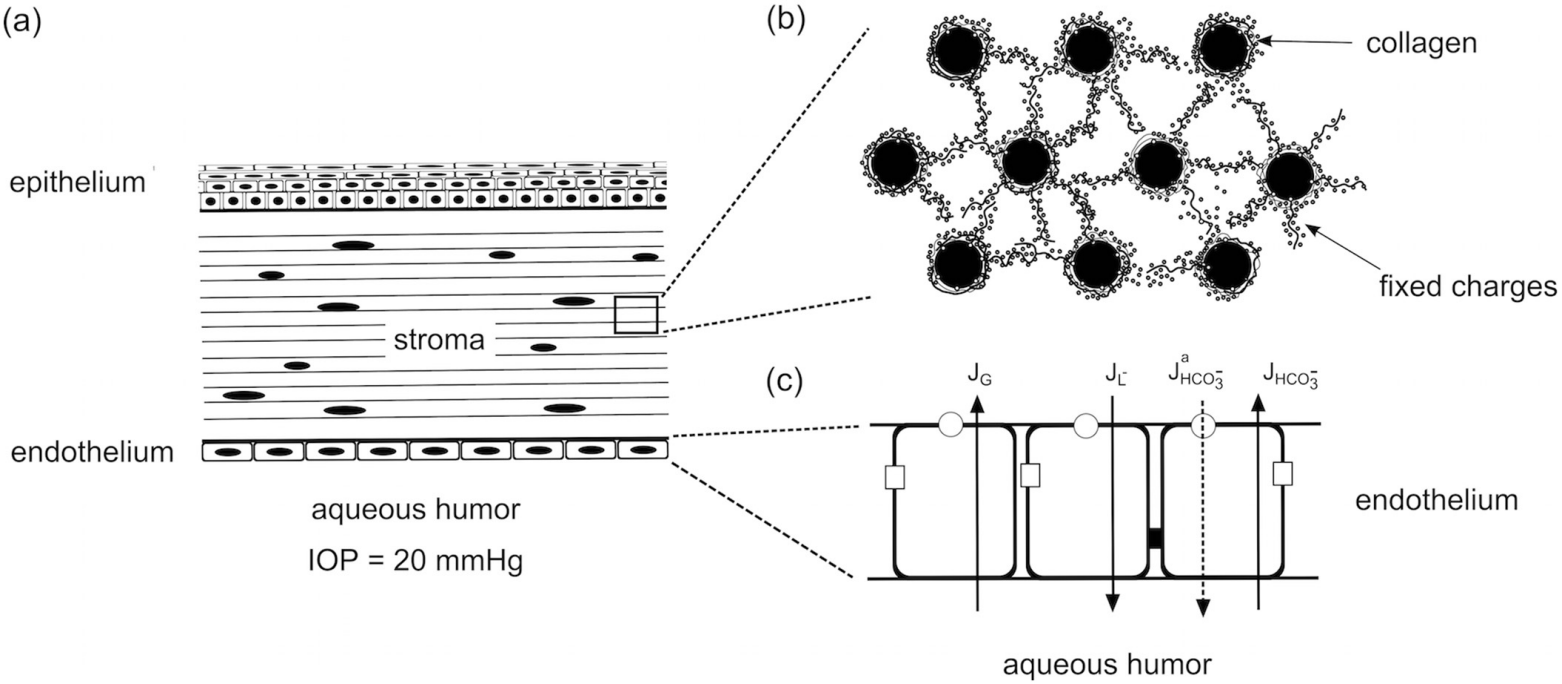
a) The cornea has three principal layers, namely the epithelium, stroma and endothelium. b) The corneal stroma is composed by collagen fibers (lamellae) packed through thickness, with keratocyte cells interspersed between adjacent lamellae. Fixed charges are associated with sulphated proteoglycans that are attached to the collagen fibrils, which form a lamella by assembling as parallel arrays following the direction of the lamella. The fixed charges give rise to a swelling tendency of the cornea [8] by the Donnan effect [17]. c) The *in vivo* cornea modulates the osmotic pressure by actively pumping ionic solutes (primarily bicarbonate) from stroma to aqueous humor [31]. In addition, glucose transports from aqueous humor to stroma for the metabolic activities needed by corneal cells, and lactate ion transports in the opposite direction [25]. The underlying molecular mechanisms of corneal endothelial pumping are still under investigation [31].

In this section an example application of Eqs (31, 40) is presented to study the endothelial transport process of the *in vivo* human cornea (see Fig 3). The exchange of fluid and ions across the endothelium controls the level of corneal hydration, which is a crucial factor for maintaining the transparency of the tissue [21, 24]. Fixed charges are associated with sulphated proteoglycans in the stroma (the bulk layer of the tissue), and generate osmotic pressure by Donnan effect [8, 27]. The active ionic transport processes located in the endothelium reduce the osmotic pressure by pumping ions out from the tissue. Furthermore, metabolic reactions take place in the *in vivo* cornea, rendering nonzero transendothelial fluxes for metabolic species (glucose, bicarbonate and lactate ions) [25, 28, 29].

At steady state, only the volume flux is zero, with the steady solute fluxes being nonzero in general. This is a more general situation than that discussed in the preceding section. We consider four ionic species, namely sodium (Na^+^), chloride (Cl^−^), bicarbonate (${\text{HCO}}_{3}^{-}$) and lactate ions (${\text{C}}_{3}{\text{H}}_{5}{\text{O}}_{3}^{-}$, denoted as L^−^), and one non-ionic species (glucose, denoted as G) distributed on both sides of the endothelium. The concentrations of these species in the outside solution (aqueous humor) are listed in table 1. The reported reflection coefficients and solute permeabilities of corneal endothelium are given in table 2. Additional model input includes the net solute fluxes for metabolic species and the active bicarbonate flux [30], which were estimated by Leung et al. [25] and are summarized in table 3. The proposed KK theory now presents a set of nonlinear equations with seven unknowns (five solute concentration + fluid pressure + electrostatic potential), which are summarized as follows:

$$\begin{array}{cc} \Delta P-\sum_{k}\left(\sigma_{k}RT\Delta C_{k}-\left(1-\sigma_{k}\right)z_{k}F{\bar{C}}_{k}\Delta \varphi \right) & =0 \end{array}$$ (48)

$$\begin{array}{cc} \omega_{i}\left(RT\Delta C_{i}+z_{i}{\bar{C}}_{i}F\Delta \varphi \right)+J_{ai} & =J_{i}\;,i={\text{Na}}^{+},{\text{Cl}}^{-},{\text{HCO}}_{3}^{-},{\text{L}}^{-},\text{G}, \end{array}$$ (49)

$$\begin{array}{cc} \sum_{k}z_{k}C_{k}+z_{\text{f}}C_{\text{f}} & =0 \end{array}$$ (50)

**Table 1:** Fields values in the aqueous humor [25].

Parameters	Value
$C_{{\text{Na}}^{+}}^{0}$ (mM)	146.55
$C_{{\text{Cl}}^{-}}^{0}$ (mM)	102.85
$C_{{\text{HCO}}_{3}^{-}}^{0}$ (mM)	36.00
$C_{L^{-}}^{0}$ (mM)	7.7
$C_{G}^{0}$ (mM)	6.9
*P*_0_ (mmHg)	20.0
*φ*_0_ (mV)	0.0

**Table 2:** Endothelial membrane properties [25].

Parameters	Value
*σ*_Na^+^_	0.45
*σ*_Cl^−^_	0.45
$\sigma_{{\text{HCO}}_{3}^{-}}$	0.38
*σ*_*L*^−^_	0.45
*σ*_*G*_	0.45
*ω*_Na^+^_ *RT*(10^−5^ cm/s)	8.0
*ω*_Cl^−^_ *RT*(10^−5^ cm/s)	8.0
$\omega_{{\text{HCO}}_{3}^{-}}RT(10^{-5}$ cm/s)	8.0
*ω*_L^−^_ *RT*(10^−5^ cm/s)	3.0
*ω*_G_ *RT*(10^−5^ cm/s)	8.0

**Table 3:** Reported solute fluxes across corneal endothelium [25].

Parameters	Value
*J*_Na^+^_	0
*J*_Cl^−^_	0
$J_{{\text{HCO}}_{3}^{-}}\left(10^{-10}\text{mol}/{\text{cm}}^{2}\cdot s\right)$	-2.12
$J_{{\text{HCO}}_{3}^{-}}^{a}\left(10^{-10}\text{mol}/{\text{cm}}^{2}\cdot s\right)$	9.4
*J*_L^−^_(10^−10^mol/cm^2^⋅*s*)	2.12
*J*_G_(10^−10^mol/cm^2^⋅*s*)	-1.71

The fluid pressure difference Δ*P* is solved as

$$\begin{array}{c} \Delta P=\sum_{i}\left(RT\Delta C_{i}+\frac{1-\sigma_{i}}{\omega_{i}}\left(J_{ai}-J_{i}\right)\right)=\Delta \Pi +P_{\sigma } \end{array}$$ (51)

where *J*_*i*_ denotes the net flow for species *i* and *P*_*σ*_ = ∑_*i*_(1−*σ*_*i*_)(*J*_*ai*_−*J*_*i*_)/*ω*_*i*_. The solute concentration difference Δ*C*_*i*_ across the endothelium can be derived as (see details in S1 Appendix B):

$$\begin{array}{c} \Delta C_{i}=-\frac{z_{i}F\Delta \varphi C_{i}^{0}+\frac{J_{ai}-J_{i}}{\omega_{i}}}{RT+\frac{1}{2}z_{i}F\Delta \varphi } \end{array}$$ (52)

where the electrostatic potential difference Δ*φ* is solved as (see in S1 Appendix B):

$$\begin{array}{c} \Delta \varphi =\frac{-B+\sqrt{B^{2}-4AC}}{2A} \end{array}$$ (53)

where parameters *A*, *B* and *C* are

$$\begin{array}{cc} A & =\frac{1}{4}z_{\text{f}}C_{\text{f}}\frac{F^{2}}{RT} \end{array}$$ (54)

$$\begin{array}{cc} B & =\frac{F}{RT}\left(RT\sum_{k}C_{k}^{0}-\sum_{k}\frac{J_{ak}-J_{k}}{2\omega_{k}}\right) \end{array}$$ (55)

$$\begin{array}{cc} C & =-RTz_{\text{f}}C_{\text{f}}+\sum_{k}\frac{z_{k}J_{ak}-z_{k}J_{k}}{\omega_{k}} \end{array}$$ (56)

**Fig 4:**
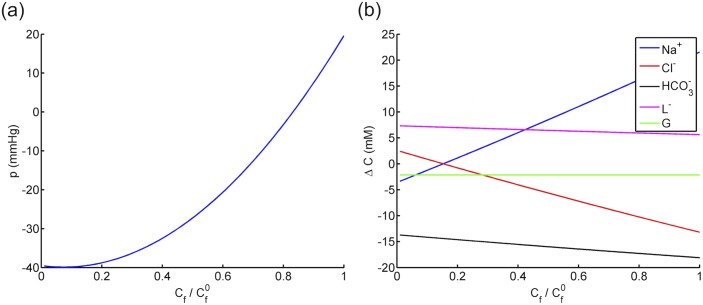
The effect of fixed charge concentration on a) the stromal uid pressure and b) the solute concentration differences across the corneal endothelium.

The effects of fixed charges on fluid pressure and solute concentrations, given by Eqs (51, 52), are illustrated in Fig 4a and 4b in the range of $C_{\text{f}}\in \left[0,C_{\text{f}}^{0}\right]$, where $C_{\text{f}}^{0}=48$ mM is the measured fixed charge concentration in corneal stroma [8]. The stromal fluid pressure *P* is predicted to be highly sensitive to *C*_f_, increasing from −39.5 to 19.6 mmHg (nearly 150% variation) as the fixed charge concentration varies from zero to $C_{\text{f}}^{0}$ (see Fig 4a). In the special case of *C*_f_ = 0 (no fixed charge), *P* is calculated to be −40 mmHg, which is comparable to the value reported by Leung et al. [25] based on the KK Eqs (5 and 6) (see table 4). The predicted sodium concentration difference Δ*C*_Na^+^_, given by Eq (52), shows the highest sensitivity to *C*_f_ among all the solutes (see Fig 4b), varying from −2.2 to +22.0 mM as *C*_f_ increases from zero to $C_{\text{f}}^{0}$ (see also table 4). On the other hand, the predicted anion concentrations (chloride, bicarbonate and lactate ions) show an opposite trend by decreasing with *C*_f_. The contrast between the curves of cation and anions is attributed to the Donnan effect, which requires accumulation of cations to compensate the concentration of negative fixed charges. As expected, the calculated glucose concentration is invariant with *C*_f_ since it is a neutral species. The calculated ionic concentrations at *C*_f_ = 0 are in close agreement with those obtained in Leung et al. [25] (see table 4).

**Table 4:** Comparisons of the solute concentrations and stationary fluid pressure at stromal-endothelial interface of the cornea by the new KK equations and that by [25].

	*C*_Na^+^_ (mM)	*C*_Cl^−^_	$C_{{\text{HCO}}_{3}^{-}}$	*C*_L^−^_	*P* (mmHg)
Leung et al. [25]	143.1	105.0	22.58	15.00	−40.5
New KK, *C*_f_ = 0	143.6	105.3	22.28	15.03	−39.5
New KK, *C*_f_ = 48.0 mM	168.5	89.67	17.91	13.33	19.6

**Fig 5:**
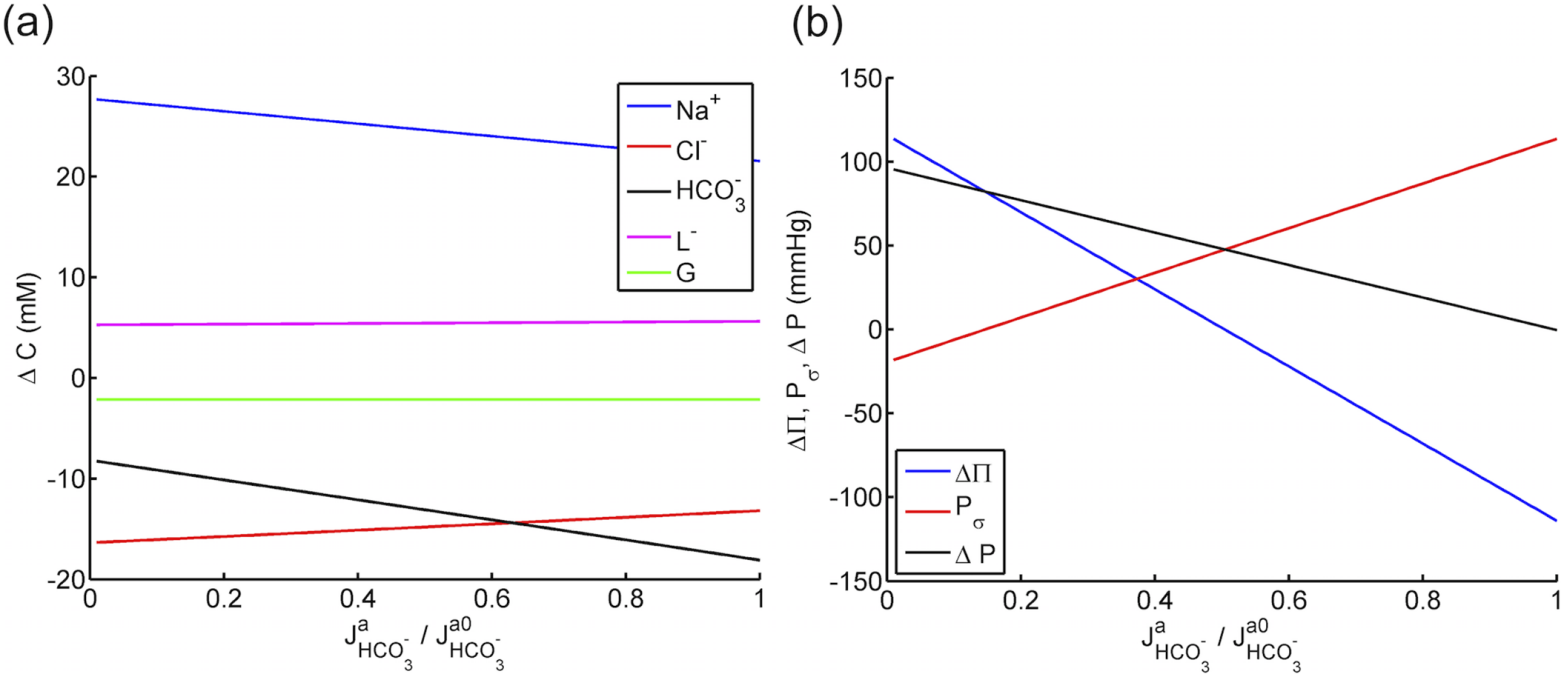
a) The predicted solute concentration differences across corneal endothelium against active bicarbonate flux. b) The predicted uid pressure difference Δ*P*, osmotic pressure difference ΔΠ and the additional pressure force *P*_*σ*_ against active bicarbonate flux for the corneal endothelium.

The effect of active bicarbonate flux $J_{{\text{HCO}}_{3}^{-}}^{a}$ on solute concentrations is presented in Fig 5a in the range of $J_{{\text{HCO}}_{3}^{-}}^{a}\in \left[0,J_{{\text{HCO}}_{3}^{-}}^{a0}\right]$, where $J_{{\text{HCO}}_{3}^{-}}^{a0}$ denotes the reported value of bicarbonate pumping flux (see table 3). Predicted bicarbonate concentration shows the most sensitivity to $J_{{\text{HCO}}_{3}^{-}}^{a}$ where $\Delta C_{{\text{HCO}}_{3}^{-}}$ varies from −10 to −20 mM as the active bicarbonate flux increases from zero to $J_{{\text{HCO}}_{3}^{-}}^{a0}$. Interestingly, the sodium concentration difference Δ*C*_Na^+^_ is also highly dependent on the bicarbonate pumping flux, reducing from 30 to about 22 mM. The chloride and lactate ion concentrations, on the other hand, increase mildly with $J_{{\text{HCO}}_{3}^{-}}^{a}$. The osmotic pressure, which is a measure of the total osmolarity including all the solutes, decreases as bicarbonate ions are pumped out from stroma to aqueous humor (see Fig 5b). The influence of $J_{{\text{HCO}}_{3}^{-}}^{a}$ on Δ*P* and *P*_*σ*_ is shown in Fig 5b. At the condition of no active transport ($J_{{\text{HCO}}_{3}^{-}}^{a}=0$), fluid pressure difference Δ*P* is predicted to be approximately 100 mmHg, a nonphysiological value that will cause the tissue to swell severely and damage the endothelial membrane [21, 30]. This result confirms the necessity of the active transport mechanism to maintain the hydration of the tissue. When $J_{{\text{HCO}}_{3}^{-}}^{a}=J_{{\text{HCO}}_{3}^{-}}^{a0}$, Δ*P* is predicted to be zero which is a result of equal osmotic pressure ΔΠ and *P*_*σ*_ on the magnitude of 120 mmHg. The two curves of ΔΠ and *P*_*σ*_ have opposite signs of slope, indicating again that the two pressure mechanisms compete with each other in generating fluid pressure in the stroma: while ΔΠ is reduced due to the active transport of bicarbonate, *P*_*σ*_ provides a countering effect by increasing with $J_{{\text{HCO}}_{3}^{-}}^{a}$. The net effect of the two mechanisms, illustrated by the curve of ΔP, shows a decreasing trend with $J_{{\text{HCO}}_{3}^{-}}^{a}$ but with a smaller slope due to *P*_*σ*_.

## Discussion

The proposed KK Eqs (31, 40) present a general framework to describe coupled transport through biological membranes. This work may be viewed as an extension from Li [16], Hodson and Earlam [18] and Hoshiko and Lindley [14] by including the effect of fixed charges, multi-component solutes (both ionic and non-ionic species) and active transport mechanisms. While the derivations follow the standard practice of the KK theory [10], the key step is to take account of the fixed charge concentration. The theory resolves the difficulties of existing KK equations in predicting transmembrane fluid pressure for charged electrolyte solution. The recovery of the fluid pressure and osmotic pressure in Donnan equilibrium provides verification of the proposed theory, and explains the swelling tendency of tissues like cornea (with fixed charge inside) with or without the presence of a bounding membrane, a phenomena that has been observed experimentally and explained qualitatively [18].

As water transport is primarily passive in biological systems [32], conventional analysis treats water as being effectively in thermodynamic equilibrium [3], with the water potential difference across membrane assumed to be zero. In this case, the osmotic pressure difference ΔΠ is balanced by the fluid pressure difference Δ*P*[21]. The analyses presented in the preceding two sections have shown that this may not be the case when active ionic transport processes are present. The generation of *P*_*σ*_ alters the pressure balance condition across membranes such that

$$\begin{array}{c} \Delta P=\Delta \Pi +P_{\sigma }\Rightarrow \mu_{w}\neq \mu_{w}^{0} \end{array}$$ (57)

This implies that the values of the water potential across the separating membrane will not be equal. According to Eq (57), the fluid pressure difference is balanced by the combined effect from ΔΠ and *P*_*σ*_. Numerically, ΔΠ and *P*_*σ*_ have opposite signs, indicating their competing effect in contributing to Δ*P*; if ions are pumped out to reduce the fluid pressure inside, the pressure *P*_*σ*_ will be positive and provide a countering effect, and vice versa. The quantitative analyses given in the preceding two sections show that *P*_*σ*_ has a significant impact on *P* when the membrane is leaky, indicated by a reflection coefficient less than unity. The significance of *P*_*σ*_ in biological system is also interesting to discuss. As active transport mechanisms take place across biological membranes to maintain the internal fluid pressure by removing ions from inside to the outside environment, the pressure force *P*_*σ*_ makes such processes less efficient—the membrane system is required to generate a stronger active flux to overcome *P*_*σ*_, which costs more energy from the active transport mechanism.

Specifically for the cornea, the pressure Eq (57) suggests a new interpretation of the stromal fluid pressure, which is an important quantity in corneal biomechanics because it interacts with the stromal collagen and modulates the fluid content of the tissue [21]. Historical views on the transendothelial fluid balance have been predicated on the “pump-leak” hypothesis [30]. It assumes negative stromal fluid pressure, which contributes a fluid leak from the aqueous humor to the stroma, and negative osmotic pressure, which results in a fluid leak in the other direction. This balancing concept can be abstracted by Δ*P* = ΔΠ, where the left hand side represents the source for the “leak” and the right hand side for the “pump.” According to Eq (57), the balancing condition can be relaxed; due to the generation of *P*_*σ*_, the stromal fluid pressure could be positive (see table 4) while the osmotic pressure is negative. This view of a positive stromal fluid pressure is corroborated by a recent structural analysis of the cornea [21], which needs further experimental verification.

It is clear from Eq (51) that *P*_*σ*_ is highly sensitive to reflection coefficient *σ*_*i*_, which is a cross coefficient that provides a measure of the interaction between the flows of solute and solvent. Although the KK theory is strictly phenomenological [9], the physical interpretation of *σ*_*i*_ is worth discussing. An alternative way of interpretating the membrane transport is to view the conjugate forces as a result of mechanical friction among solute (water) and solutes [33]. Accordingly, *σ*_*i*_ has been found to be governed by the solute permeability through the membrane and the friction between solvent and solute. It will be unity only if the membrane is strictly impermeable to solute species *i*. On the contrary, the more leaky the membrane is to solutes and the stronger the interactions between the solute and solvent phases, the smaller *σ*_*i*_ will be, which results in a larger *P*_*σ*_ as shown in the current analysis.

Extensions on the current work include two aspects. First, the new KK theory can be incorporated into continuum models for cells or tissues to describe the interactions between membrane transport and the bulk electrolyte inside. A multiphasic model for the living cornea that accounts for hydration, fixed charges and endothelial transport is under development. Second, it should be noted that the assumption of an independently specified active ionic flux in the above theory, for example $J_{{\text{HCO}}_{3}^{-}}^{a}$ in the preceding section, is employed for simplicity [24]. The consideration of active ionic flux, and its coupling with the solvent and solutes can be elaborated by taking into account the underlying metabolic reactions [9]. This development would require identification of the molecular mechanisms, but could potentially enhance the understanding of active mechanism involvement in membrane transport.

## Supporting Information

S1 Appendix Derivations of the KK equations considering active transport and determinations of fluid pressure and solute concentrations at steady state. (PDF)
