# Supplementary material for: The Balance of Fluid and Osmotic Pressures across Active Biological Membranes with Application to the Corneal Endothelium
Source: PLoS One. 2015 Dec 31;10(12):e0145422. doi: 10.1371/journal.pone.0145422 (PMC4697791; doi:10.1371/journal.pone.0145422)
Supplement: S1 Appendix — (PDF) [file pone.0145422.s001.pdf]

S1 Appendix for "The balance of fluid and osmotic pressures across active biological membranes with application to the corneal endothelium"

Xi Cheng and Peter M. Pinsky

September 18, 2015

A Derivation of the KK equations with incorporation of active transport

Consider the coupled transport through a leaky membrane when M metabolic reactions take place to actively transport ions. The dissipation function of the system is given as [1]

$$\Phi = J_w X_w + \sum_{i=1}^{N} J_i X_i + \sum_{j=1}^{M} J_{r_j} A_j \tag{A.1}$$

where J_{r_j} and A_j are the reaction rate and affinity of the jth reaction. For a reaction expressed as:

$$\nu_B B + \nu_C C \rightarrow \nu_D D + \nu_E E \tag{A.2}$$

where the $\nu's$ are the stoichiometric coefficients of the reaction in which the reactants B, C are converted to the products C, D. The thermodynamic definition of the above reaction's affinity is

$$A = -\sum_i \nu_i \mu_i = -\nu_B \mu_B - \nu_C \mu_C - \nu_D \mu_D - \nu_E \mu_E \tag{A.3}$$

where $\mu's$ are the electrochemical potential defined as eq. (12). Following the transformations shown in section 3, we can express the volume flux J_V, the exchange flux $J_{D_i}, i = 1, ..., N$ and the reaction rate $J_{r_j}, j = 1, ...M$ as linear combinations of the conjugate forces $\Delta\widetilde{P}$, $\Delta\widetilde{\Pi}_i$ and A_j, respectively. For conveniences of adding the reaction rate J_{r_j} in the volume flux and

exchange flux expressions [1], an alternative form of J_V, J_{D_i}, J_{r_j} can be written as:

$$J_V = L_{VV}\Delta\widetilde{P} + \sum_{k=1}^{N} L_{Vk}\Delta\widetilde{\Pi}_k + \sum_{j=1}^{M} L_{Vr_j} J_{r_j} \tag{A.4}$$

$$J_{D_i} = L_{iV}\Delta\widetilde{P} + \sum_{k=1}^{N} L_{D_ik}\Delta\widetilde{\Pi}_k + \sum_{j=1}^{M} L_{D_ir_j} J_{r_j} \tag{A.5}$$

$$J_{r_i} = L_{r_iV}\Delta\widetilde{P} + \sum_{k=1}^{N} L_{r_iD_k}\Delta\widetilde{\Pi}_k + \sum_{j=1}^{M} L_{r_ir_j} A_j \tag{A.6}$$

in which the coefficients are not completely independent. The coefficient $L_{Vr_j}, L_{D_ir_j}$ and $L_{r_ir_j}$ are defined as

$$L_{Vr_j} = \left(\frac{\partial J_V}{\partial J_{r_j}}\right)_{\Delta\widetilde{P},\Delta\widetilde{\Pi}_1,\ldots,\Delta\widetilde{\Pi}_N} \tag{A.7}$$

$$L_{D_ir_j} = \left(\frac{\partial J_{D_i}}{\partial J_{r_j}}\right)_{\Delta\widetilde{P},\Delta\widetilde{\Pi}_1,\ldots,\Delta\widetilde{\Pi}_N} \tag{A.8}$$

$$L_{r_ir_j} = \left(\frac{\partial J_{r_i}}{\partial A_j}\right)_{\Delta\widetilde{P},\Delta\widetilde{\Pi}_1,\ldots,\Delta\widetilde{\Pi}_N} \tag{A.9}$$

the other two set of cross coefficients can be determined by the Onsager reciprocal relationships as:

$$\left.\frac{\partial J_V}{\partial A_i}\right|_{\Delta\widetilde{P},\Delta\widetilde{\Pi}_1,\ldots,\Delta\widetilde{\Pi}_N} = \left.\frac{\partial J_{r_i}}{\partial \Delta\widetilde{P}}\right|_{\Delta\widetilde{\Pi}_1,\ldots\Delta\widetilde{\Pi}_N,A_1,\ldots,A_M}$$

$$\Rightarrow L_{r_iV} = \sum_{j=1}^{M} L_{r_jr_i} L_{Vr_j} \tag{A.10}$$

$$\left.\frac{\partial J_{D_i}}{\partial A_j}\right|_{\Delta\widetilde{P},\Delta\widetilde{\Pi}_1,\ldots,\Delta\widetilde{\Pi}_N} = \left.\frac{\partial J_{r_j}}{\partial \Delta\widetilde{\Pi}_i}\right|_{\Delta\widetilde{P},A_1,\ldots,A_M}$$

$$\Rightarrow L_{r_jD_i} = \sum_{k=1}^{M} L_{D_ir_k} L_{r_kr_j} \tag{A.11}$$

(A.12)

Define the hydraulic coefficient and reflection coefficients as:

$$L_p = L_{VV} = \left(\frac{\partial J_V}{\partial \Delta\widetilde{P}}\right)_{\Delta\widetilde{\Pi}_j,J_{r_k}} \tag{A.13}$$

$$\sigma_i = -\frac{L_{Vi}}{L_{VV}} \tag{A.14}$$

and simplify the notations as

$$L_{Vr_j} = V_j, j = 1, ..., M \tag{A.15}$$

$$L_{D_i r_j} = U_{ij}, i = 1, ..., N, j = 1, ..., M \tag{A.16}$$

$$L_{r_i r_j} = K_{ij}, i, j = 1, ..., M \tag{A.17}$$

The flux equations are then then written as:

$$J_V = L_p \Delta\widetilde{P} - L_p \sum_{k=1}^{N} \sigma_k \Delta\widetilde{\Pi}_k + \sum_{j=1}^{M} V_j J_{r_j} \tag{A.18}$$

$$J_{D_i} = -L_p \sigma_i \Delta\widetilde{P} + \sum_{k=1}^{N} L_{D_i k} \Delta\widetilde{\Pi}_k + \sum_{j=1}^{M} U_{ij} J_{r_j} \tag{A.19}$$

$$J_{r_i} = \sum_{j=1}^{M} K_{ji} V_j \Delta\widetilde{P} + \sum_{j=1}^{M}\sum_{k=1}^{N} U_{kj} K_{ji} \Delta\widetilde{\Pi}_j + \sum_{j=1}^{M} K_{ij} A_j \tag{A.20}$$

Finally, we utilize eq. (29) and solve the solute fluxes as

$$J_i = (1-\sigma_i)\overline{C}_i J_V + \sum_{k=1}^{N} \omega_{ik} \Delta\tilde{\Pi}_k + \overline{C}_i \left(\sum_{j=1}^{M} (\sigma_i V_j + U_{ij}) J_{r_j} \right) \tag{A.21}$$

where $\omega_{ik} = \overline{C}_i (L_{D_i k} - L_p \sigma_i \sigma_k)$. Ignoring the coupling between ionic fluxes and between different reactions, i.e, $\omega_{ij} = \omega_i \delta_{ij}$ and $K_{ij} = K_i \delta_{ij}$, the new flux equations can be summarized as:

$$J_V = L_p \left(\Delta\tilde{P} - \sum_{k=1}^{N} \sigma_k \Delta\tilde{\Pi}_k \right) + \sum_{j=1}^{M} V_j J_{r_j} \tag{A.22}$$

$$J_i = (1-\sigma_i)\overline{C}_i J_V + \omega_i \Delta\tilde{\Pi}_i + \overline{C}_i \left(\sum_{j=1}^{M} (\sigma_i V_j + U_{ij}) J_{r_j} \right) \tag{A.23}$$

$$J_{r_i} = K_i \left(V_i \Delta\tilde{P} + \sum_{k=1}^{N} U_{ki} \Delta\tilde{\Pi}_k + A_i \right) \tag{A.24}$$

B Determination of pressure, ionic concentrations and electrostatic potential in the new KK theory

Consider the KK equations (31, 40) for a general trans-endothelial diffusion process as described in section 5. For stationary state where $J_V = 0$, the KK equations can be rewritten as:

$$\Delta P - \sum_k \left(\sigma_k RT \Delta C_k - (1-\sigma_k) z_k F \overline{C}_k \Delta\varphi \right) = 0 \tag{B.1}$$

$$RT\Delta C_i + z_i \overline{C}_i F \Delta\varphi + \frac{J_{ai} - J_i}{\omega_i} = 0 \quad , i = 1, ..., N \tag{B.2}$$

where $\overline{C}_i = (C_i + C_i^0)/2$ denotes the mean solute concentration across the membrane. Given $J_V, J_i,\ J_{ai}(i = 1, ..., N)$ and the fixed charge concentration C_f, the fluid pressure difference ΔP, ionic concentration ΔC_i and electrostatic potential $\Delta\varphi$ can be solved by combining the above equations with the electroneutrality condition

$$\sum_k z_k C_k + z_\mathrm{f} C_\mathrm{f} = 0 \tag{B.3}$$

To solve the nonlinear set of equations (B.1 - B.3), we first solve for $\Delta\varphi$. Combining the N equations in (B.2) gives:

$$\Pi - \Pi^0 - \frac{1}{2} z_\mathrm{f} C_\mathrm{f} F \Delta\varphi + \sum_k \frac{J_{ak} - J_k}{\omega_k} = 0 \tag{B.4}$$

where $\Pi^0 = RT \sum_k C_k^0$ and $\Pi = RT \sum_k C_k$ denote the osmotic pressures of the two electrolytes separated by the membrane, respectively. Similarly, combining the product of z_i and the N equations in (B.2) gives:

$$-RT z_\mathrm{f} C_\mathrm{f} + \frac{F}{2RT}(\Pi^0 + \Pi)\Delta\varphi + \sum_k \frac{z_k J_{ak} - z_k J_k}{\omega_k} = 0 \tag{B.5}$$

where the electroneutrality condition of the outside electrolyte, i.e. $\sum_k z_k C_k^0 = 0$, has been used in eqs. (B.4, B.5). From eq. (B.4) Π is expressed as:

$$\Pi = \Pi^0 + \frac{1}{2} z_\mathrm{f} C_\mathrm{f} F \Delta\varphi - \sum_k \frac{J_{ak} - J_k}{\omega_k} \tag{B.6}$$

substituting eq.(B.6) into eq.(B.5) gives:

$$\frac{1}{4} z_\mathrm{f} C_\mathrm{f} \frac{F^2}{RT} \Delta\varphi^2 + \frac{F}{RT}\left(\Pi^0 - \sum_k \frac{J_{ak} - J_k}{2\omega_k}\right)\Delta\varphi - RT z_\mathrm{f} C_\mathrm{f} + \sum_k \frac{z_k J_{ak} - z_k J_k}{\omega_k} = 0 \tag{B.7}$$

The above equation is a quadratic equation of the electrostatic potential $\Delta\varphi$, which is solved as:

$$\Delta\varphi = \frac{-B \pm \sqrt{B^2 - 4AC}}{2A} \tag{B.8}$$

where parameters A, B and C are

$$A = \frac{1}{4} z_\mathrm{f} C_\mathrm{f} \frac{F^2}{RT} \tag{B.9}$$

$$B = \frac{F}{RT}\left(\Pi^0 - \sum_k \frac{J_{ak} - J_k}{2\omega_k}\right) \tag{B.10}$$

$$C = -RT z_\mathrm{f} C_\mathrm{f} + \sum_k \frac{z_k J_{ak} - z_k J_k}{\omega_k} \tag{B.11}$$

depending on the value of z_f, one of the root in eq. (B.8) is eliminated for giving negative ionic concentration. For the application in section 5, $\Delta\varphi = (-B + \sqrt{B^2 - 4AC})/2A$. Substitute φ into eq. (B.2), the solute concentration C_i is solved as:

$$C_i = \frac{RTC_i^0 - \frac{1}{2} z_i F C_i^0 \Delta\varphi - \frac{J_{ai} - J_i}{\omega_i}}{RT + \frac{1}{2} z_i F \Delta\varphi} \tag{B.12}$$

References

[1] Hoshiko T, Lindley BD, 1967. Phenomenological description of active transport of salt and water. J. Gen. Physiol. 50, 729-758.
